# Supplementary material for: Predicting the genetic component of gene expression using gene regulatory networks
Source: Bioinform Adv. 2024 Nov 23;4(1):vbae180. doi: 10.1093/bioadv/vbae180 (PMC11665636; doi:10.1093/bioadv/vbae180)
Supplement: vbae180_Supplementary_Data [file vbae180_supplementary_data.pdf]

Predicting the genetic component of gene expression using gene regulatory networks

Supplementary Information

Gutama Ibrahim Mohammad[1] and Tom Michoel[1,*]

[1] Computational Biology Unit, Department of Informarics, University of Bergen, Norway
[*] Corresponding author, email: tom.michoel@uib.no

Contents

S1 Gene Expression Prediction Algorithm Using Gene Regulatory Networks **2**

S2 Data processing details **3**
- S2.1 DREAM5 data . . . 3
- S2.2 Geuvadis data . . . 3

S3 Comparison of prediction methods **4**

S4 Correlation Clustermap Analysis **5**
- S4.1 DREAM Dataset . . . 5
- S4.2 Yeast Dataset . . . 5
- S4.3 Geuvadis Dataset . . . 6

S5 Network Statistics **8**

S6 Using All cis-eQTLs Geuvadis data **8**
- S6.1 Comparison of prediction methods . . . 8
- S6.2 Relevance of network information . . . 9

S1 Gene Expression Prediction Algorithm Using Gene Regulatory Networks

Algorithm 1 Gene Expression Prediction using a Gene Regulatory Network.

Require: G - Directed Acyclic Graph representing Gene Regulatory Network
Require: E - Genotype-matrix, genotype of cis-eQTLs associated with each gene in G from N peoples (available in public database)
Require: X - Expression value matrix for all genes in G, from N same sample as in E
Ensure: $\hat{X}$ - Empty dictionary to store predicted value of each gene in G
Require: $Prediction\_Model$ - A machine learning model to use for prediction.
Require: $UsePredictedExpression$ - A boolean flag to decide whether to use predicted expressions in subsequent predictions.

```
function IsRootNode(A, H)
    return True if g_i has no incoming edges in G else False
end function
function PredictGeneExpression(G, E, X, UseParentGenotype, UseGrandParentGenotype, UsePre
    Validate that G is a DAG and handle errors
    SortedNodes ← Topologically sort nodes in G
    X̂ ← {} Initialize predicted expressions
    for i, g_i in enumerate(SortedNodes) do
        E_i ← Genotype of eQTLs of gene g_i from E
        E_i^p ← Genotypes of eQTLs of parent genes to g_i from E
        E_i^gp ← Genotypes of eQTLs of both parents and grand parents of g_i from E
        X_i^p ← Expression of parents of g_i from X
        Input_cis ← E_i
        X̂_i^cis ← Prediction_Model(Input_cis)
        if IsRootNode(g_i, G) then
            X̂[i] ← X̂_i^cis
        else
            if UseParentGenotype then
                Input_trans ← E_i^p
            else if UseGrandParentGenotype then
                Input_trans ← E_i^pp
            else
                Input_trans ← X_i
            end if
        end if X̂_i^trans ← Prediction_Model(Input_trans)
        X̂[i] ← X̂_i^cis + X̂_i^trans
    end for
    if UsePredictedExpression then
        PredictGeneExpression(G, E, X̂, False, False, True)
    end if
    return Prediction_Model
end function
```

S2 Data processing details

S2.1 DREAM5 data

The DREAM5 Systems Genetics Challenge A consisted of 15 sub-datasets, each representing a different network. The sub-datasets were created through simulations involving different sample sizes (100, 300, and 999) across five distinct networks. Each network consisted of 1000 genes and their corresponding genotypes. In each sub-dataset, there was a one-to-one correspondence between each gene and a genotype variable, with 25% of these genotype variables being cis-expression Quantitative Trait Loci (eQTL). For our analysis, we utilized the first 1000 gene network and dataset with 1000 samples.

To identify the 250 cis-eQTLs we used the Kruskal-Wallis test to get pvalues and selected the top 25% based on the strength of association between gene expression and genetic variants.

The dataset is available at https://www.synapse.org/

S2.2 Geuvadis data

We used the PLINK2 software with the "–export Av" flag to convert biallelic SNP genotypes to a matrix of (0/1/2/'NA')-values, representing the major homozygous genotype, the heterozygous genotype, the minor homozygous genotype, and missing data, respectively.

We removed any SNPs that had missing data in one or more samples. Additionally, we discarded SNPs where the minor allele frequency (MAF) was less than 5% of the total allele frequency. In practice, multiple genes often share the same SNP as their most significant cis-eQTL. However, we also removed these genes due to warnings from the network reconstruction tool we are using. After removing SNPs that are the most significant eQTL for multiple genes, we obtained 2979 genes, each with one unique most significant cis-eQTL.

It is worth noting that although each gene is linked to at least one SNP, the majority of genes are associated with multiple SNPs. In order to reconstruct the gene regulatory network, we only took into account the most significant cis-eQTL for each gene (because our network reconstructiion tool requires one eQTL per gene). However, when training the gene prediction models, we can consider all eQTLs for each gene.

S3 Comparison of prediction methods

A. DREAM

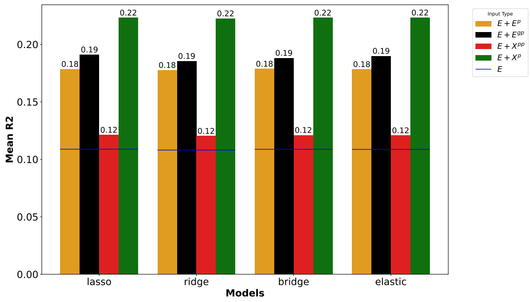

B. Yeast

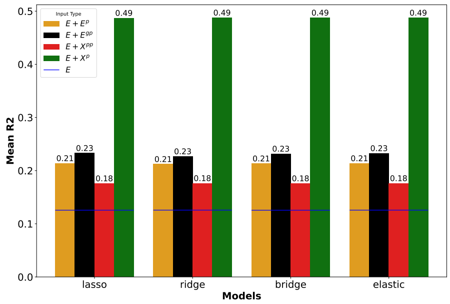

C. Geuvadis

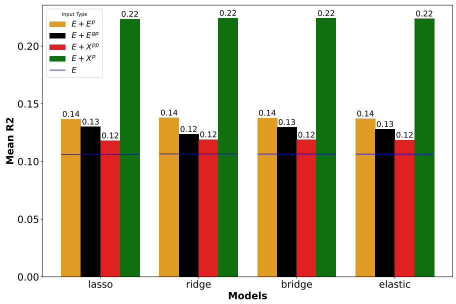

Figure S1: **Comparison of Mean $R2$ Values for Different Models Across DREAM, Yeast, and Geuvadis Datasets**. The figures present the mean R values (over GRN) for various regularized regression models predicting gene expression levels within Gene Regulatory Networks (GRNs) for three datasets: DREAM (A), Yeast (B), and Geuvadis (C). Each figure displays the performance of Lasso, Ridge, Bridge, and Elastic Net models using different combinations of input features: cis-eQTLs alone (E), cis-eQTLs combined with parent eQTLs (E + Ep), parents and grandparents eQTLs (E + Egp), actual gene expression levels of parents (E + Xp), and recursively predicted parent expression levels (E + Xpp).

S4 Correlation Clustermap Analysis

S4.1 DREAM Dataset

Figure S2 presents the gene correlation heatmaps with hierarchical clustering for the DREAM dataset. The gene correlation heatmap for the DREAM dataset shows predominantly weak red shades, indicating weak positive correlations among genes. The hierarchical clustering suggests some grouping, but the clusters are not very distinct. This pattern suggests a more stochastic or random interaction among the genes, implying that the gene interactions in this dataset are not highly structured.

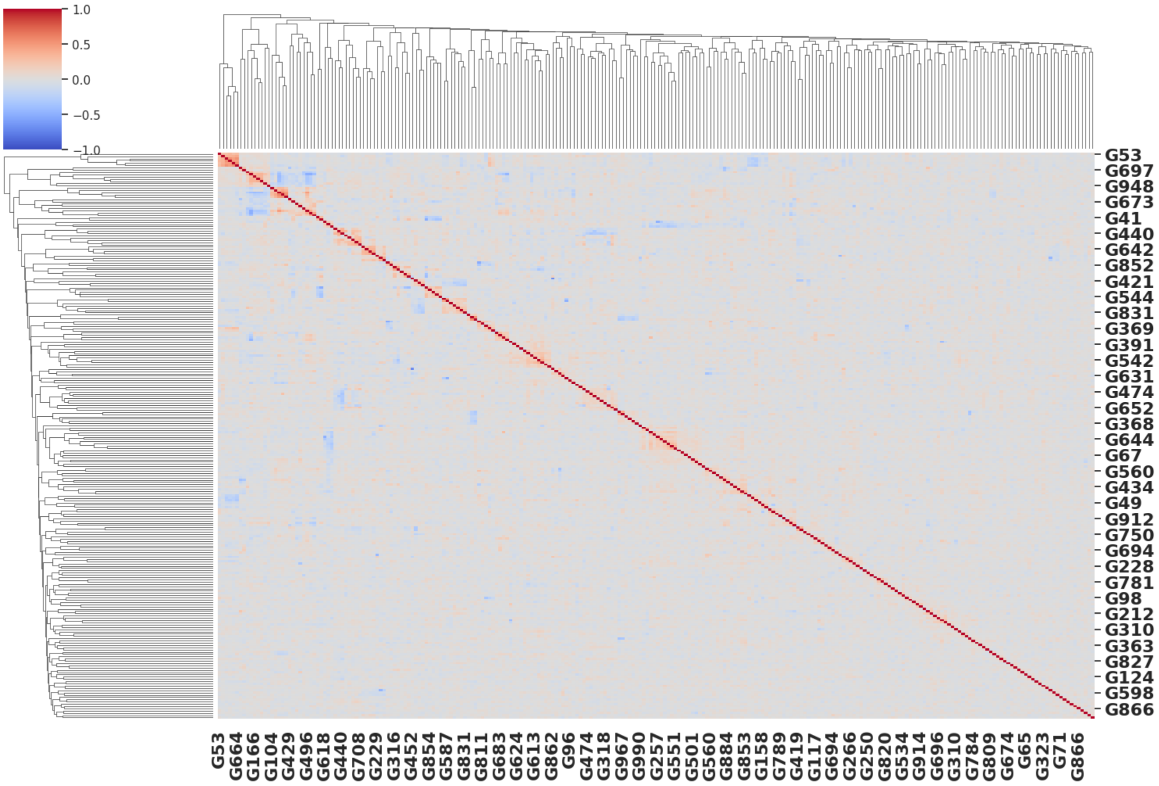

Figure S2: **Gene Correlation Heatmap Clustermap for the DREAM Dataset**. This heatmap illustrates the correlation between different genes in the DREAM dataset, with hierarchical clustering applied to group genes with similar correlation patterns. The predominantly weak red shading indicates a lack of strong gene interactions, and the clustering reveals a few less distinct clusters, suggesting a stochastic pattern of gene interactions..

S4.2 Yeast Dataset

Figure S3 shows the gene correlation heatmaps with hierarchical clustering for yeast data. The gene correlation heatmap for the Yeast dataset displays a clear pattern with strong positive (red) and negative (blue) correlations among genes. The hierarchical clustering reveals well-defined clusters, indicating groups of genes that have highly correlated expression profiles. This structured interaction implies a high degree of correlation organization within the yeast

dataset, with certain genes interacting closely within specific clusters.

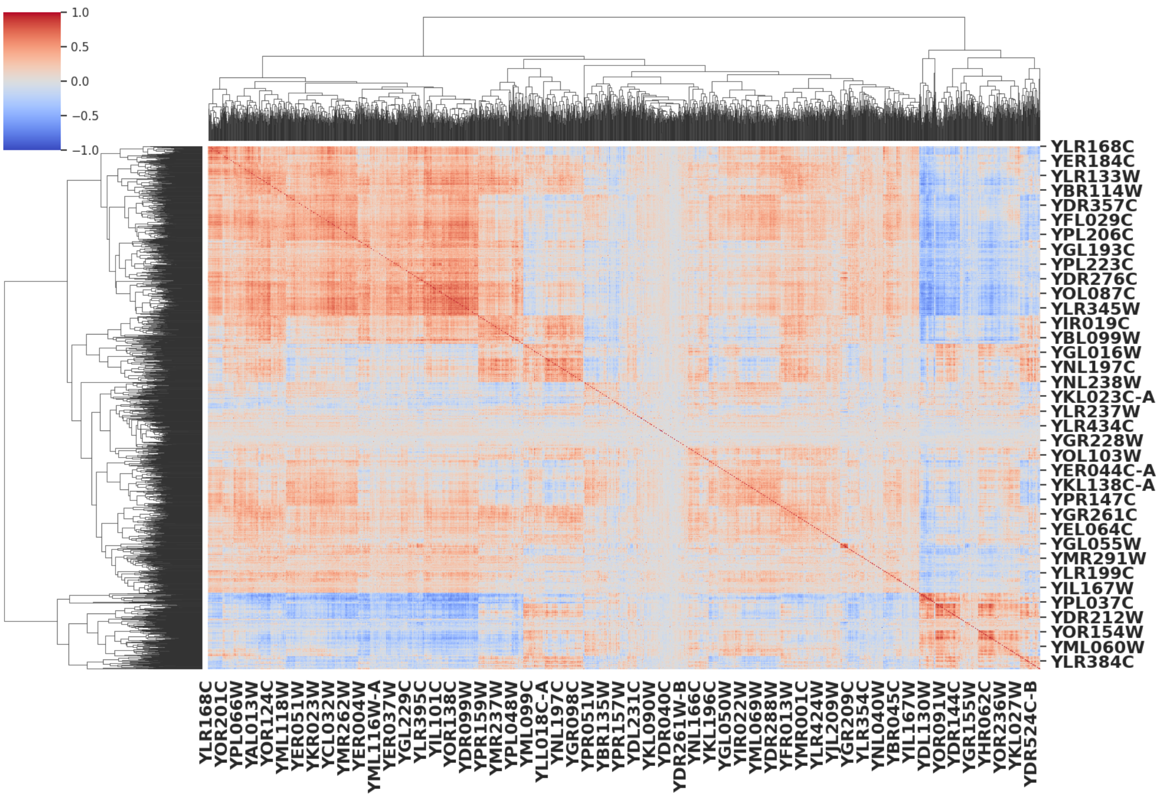

Figure S3: **Gene Correlation Heatmap Clustermap for the Yeast Dataset**. This heatmap shows the correlation between genes in the Yeast dataset, with hierarchical clustering highlighting groups of genes with similar correlation profiles. The clear patterns of positive (red) and negative (blue) correlations, along with well-defined clusters, indicate a high degree of structured gene interactions.

S4.3 Geuvadis Dataset

Figure S4 shows the gene correlation heatmaps with hierarchical clustering for Geuvadis data.The gene correlation heatmap for the Geuvadis dataset shows mostly weak red shades, indicating weak positive correlations. The hierarchical clustering reveals some clusters, but these clusters have more diffuse boundaries compared to the yeast dataset. This suggests that while there are some groups of correlated genes, the overall interaction structure is less pronounced than in yeast. The gene interactions in the Geuvadis dataset are less structured, indicating weaker regulatory relationships.

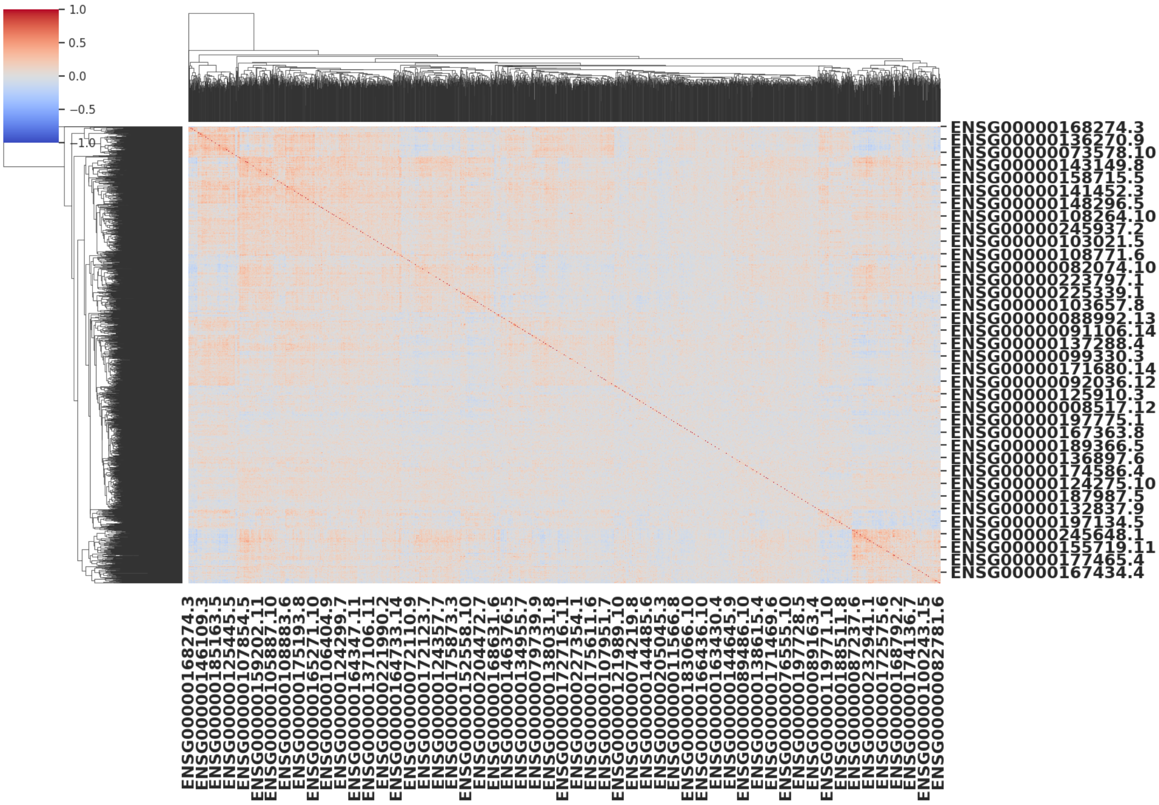

Figure S4: **Gene Correlation Heatmap Clustermap for the Geuvadis Dataset**. This heatmap displays the correlation among genes in the Geuvadis dataset, with hierarchical clustering to identify clusters of genes with correlated expression. The predominantly weak red shading suggests weaker positive correlations and less structured gene interactions. The hierarchical clustering reveals clusters with more diffuse boundaries.

S5 Network Statistics

(a) DREAM

	p	p2p5	p0
edge posterior	0.65	0.4	0.97
global fdr	0.21	0.33	0.007
total nodes	215	204	211
num root nodes	41	43	16
num leaf nodes	50	50	70
num intermidate node	124	111	125
total edges	543	447	706

(b) Yeast

	p	p2p5	p0
edge posterior	0.9997	0.9994	0.99995
global fdr	0.0002	0.0004	0.000002
total nodes	2107	2105	2399
num root nodes	495	489	337
num intermidate nodes	1164	1152	1994
num leaf nodes	448	464	68
total edges	13888	13861	186008

(c) Geuvadis

	p	p2p5	p0
edge posterior	0.7	0.5	0.9996
global fdr	0.23	0.36	0.0002
total nodes	2495	2087	1887
num root nodes	81	93	474
num intermidate nodes	684	472	1095
num leaf nodes	1730	1522	317
total edges	9018	5028	16020

Table S1: Network Statistics Comparison for (a) DREAM5 data, (B) Yeast data and (c) Geuvadis Data

S6 Using All cis-eQTLs Geuvadis data

S6.1 Comparison of prediction methods

Figure S5 shows the performance of different regularized regression models (Lasso, Ridge, Bridge, and Elastic Net) on the Geuvadis dataset using various inputs, considering all cis-eQTLs rather than only the most significant eQTL. This approach aims to provide a more comprehensive understanding of the predictive power of all cis-eQTLs on low sample dataset. The mean $R2$ values for each combination of model and input type are compared to understand the influence of different input types on prediction accuracy.

The baseline model (E) have approximately 0.85 across all models (Lasso, Ridge, Bridge, Elastic Net).

Mean $R2$ of Parent eQTLs $(E + E^p)$ slightly better than the baseline, around $0,11$ for all models. The inclusion of parent eQTLs does n improve performance, indicating that for this dataset, parent eQTLs provide some additional predictive power when using all cis-eQTLs.

Mean $R2$ of Grandparent eQTLs $(E + E^{gp})$, between $0.08 - 0.09$ for all models. Meaning adding grandparent eQTLs do not enhance prediction accuracy in this dataset. Probabilily, due to overfitting due to large number of features.

The results suggest that while cis-eQTLs provide a solid foundation for gene expression prediction, the addition of parent and grandparent eQTLs with all cis eQTL does not contribute significantly in the when sample size is low.

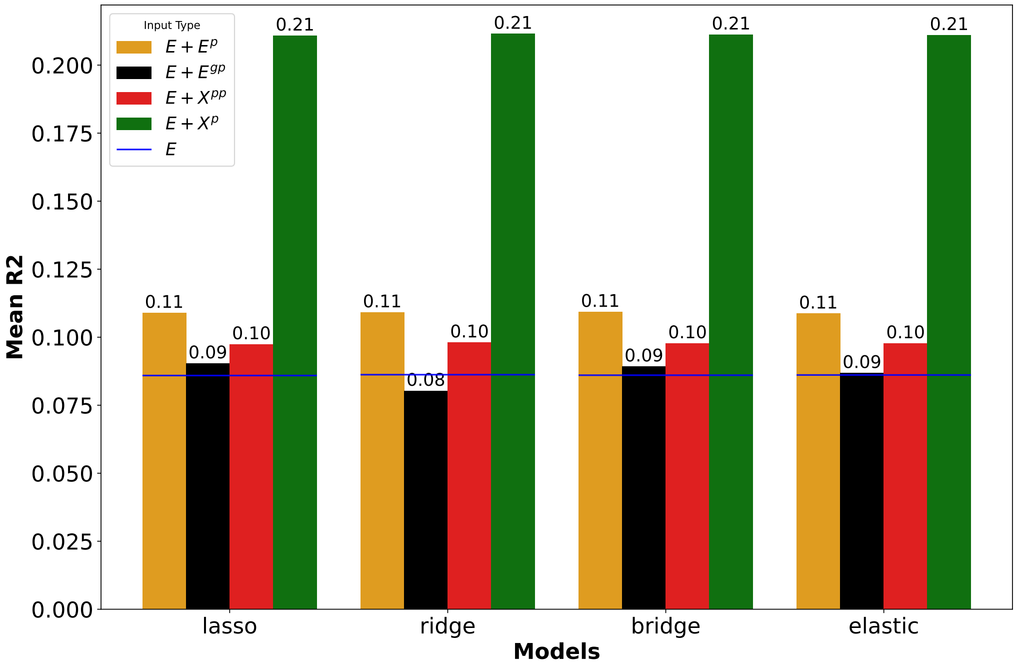

Figure S5: Comparison of Mean $R2$ Values for Different Regularized Regression Models and Input Types on the Geuvadis Dataset Using All Cis-eQTLs

S6.2 Relevance of network information

Figure S6 presents the performance of gene expression prediction models on the Geuvadis dataset using different input types, where we use all eQTLs. The networks analyzed include the best causal network (P) and (P2P5), and a correlation network (P0). The mean $R2$ values are compared across these networks to understand the impact of different inputs and networks on prediction accuracy.

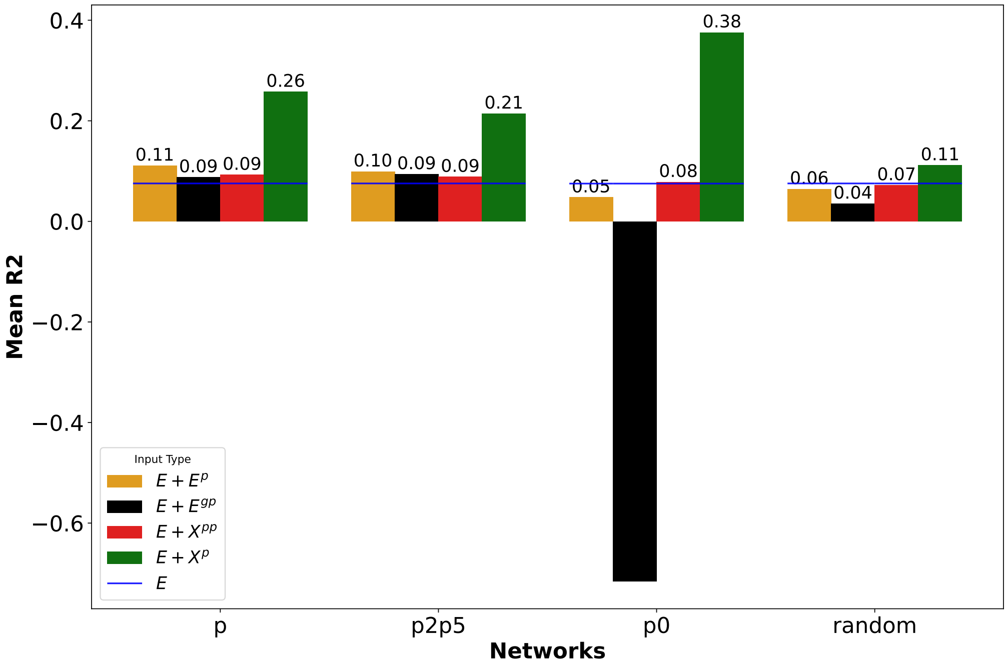

Figure S6: Comparison of Mean $R2$ Values for Different Networks and Input Types on the Geuvadis Dataset Using All Cis-eQTLs

Figure S7 shows the performance of different gene expression prediction models on the Geuvadis dataset using three different input types: cis-eQTLs (E), parent eQTLs ($E + E^p$), and grandparent eQTLs ($E + Egp$). The networks analyzed include the best causal network (P) and (P2P5), and a correlation network (P0). The pair grid results provide a direct comparison of predictive performance for each pair of networks on the same genes and inputs by showing $R2$ values for each predicted gene.

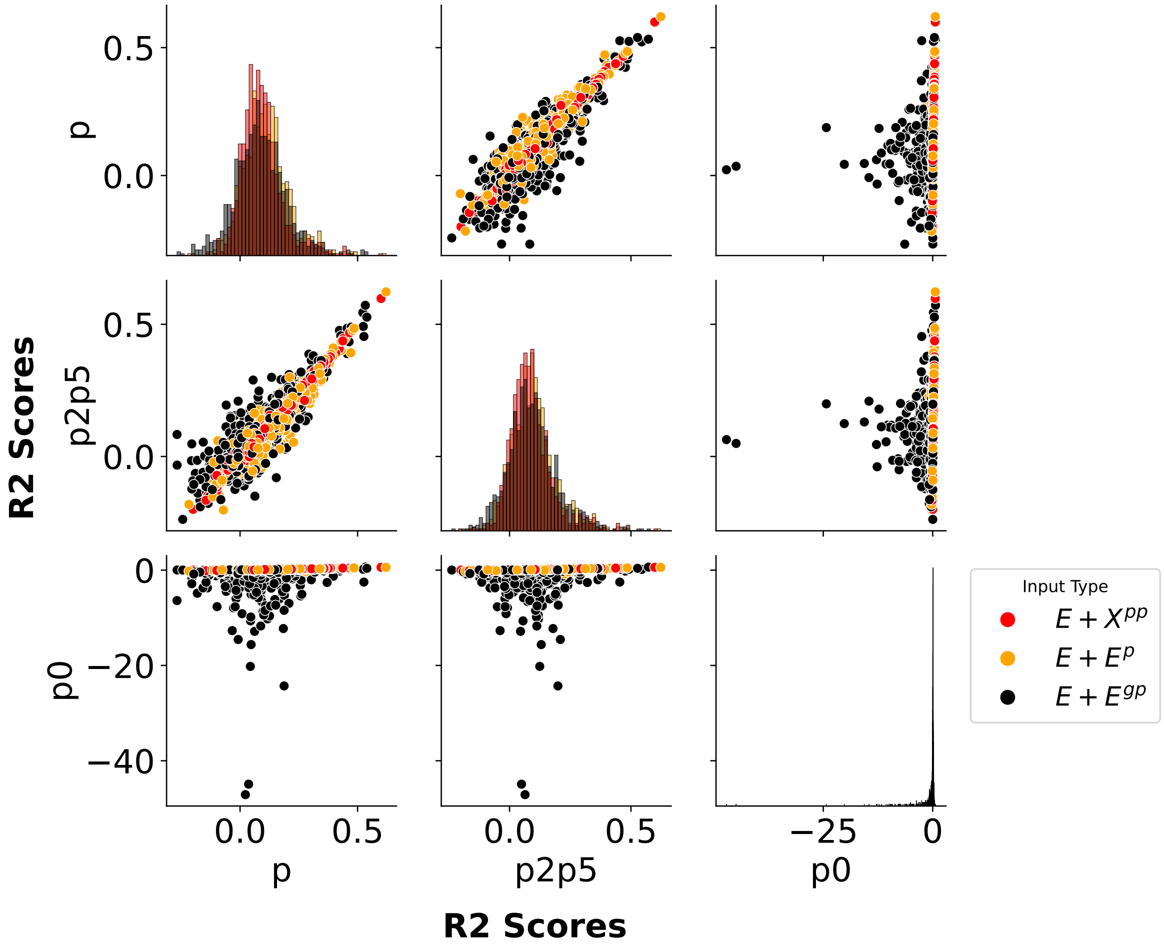

Figure S7: Pair Grid Comparison of $R2$ Values for Different Networks and Input Types on the Geuvadis Dataset Using All Cis-eQTLs

A. DREAM

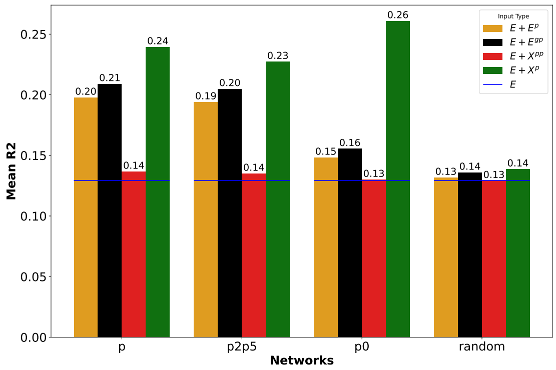

B. Yeast

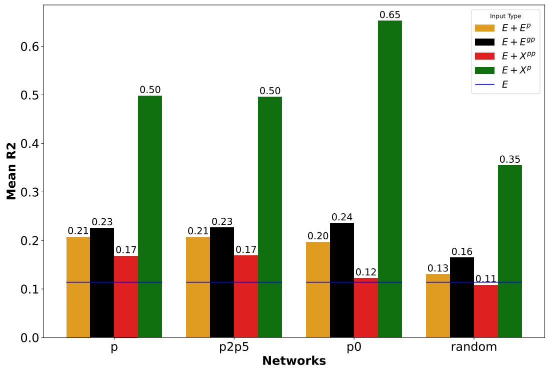

C. Geuvadis

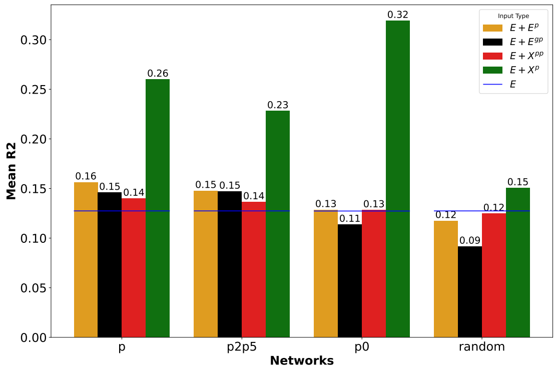

Figure S8: **Assessment of the importance of network information.** Mean R^2 scores are shown of Bayesian Ridge regression using four reconstructed GRNs using Findr and five random networks (aggregated for plotting purposes), on DREAM data (**A**) and Geuvadis (**B**) data.
